# Supplementary material for: Viral and immune dynamics of genital human papillomavirus infections in young women with high temporal resolution
Source: PLoS Biol. 2025 Jan 21;23(1):e3002949. doi: 10.1371/journal.pbio.3002949 (PMC11750104; doi:10.1371/journal.pbio.3002949)
Supplement: S1 Supplementary Materials — (PDF) [file pbio.3002949.s001.pdf]

Supplementary Materials

A Supplementary Results

A.1 Cohort profile stratified by focal HPV infection status

Table A. PAPCLEAR cohort profile stratified by HPV focal infection status. Significant differences according to a t-test with a 5% threshold are shown in bold font (unadjusted p-values). Table 1 shows the same variables stratified by HPV status. Focal infections are defined as two consecutive visits for the same HPV. Participants with a transient infection are not included in this analysis (see Methods and Fig O). For time-varying variables (BMI, Stress level and all variables identified with 'last 2 weeks'), only the inclusion visit was considered.

	No focal infection	One or more focal infections	p
number of participants (n)	97	92	
**Lifetime number of partners (mean (SD))**	8.82 (8.21)	13.16 (11.87)	0.004
**Vaccinated against HPV = Yes (%)**	57 (58.8)	40 (43.5)	0.050
Age at first visit (mean (SD))	21.63 (1.99)	21.46 (2.05)	0.559
**Age at menarchy (mean (SD))**	12.54 (1.34)	12.97 (1.32)	0.028
**Duration of follow up (days) (mean (SD))**	195.91 (180.04)	329.27 (217.27)	¡0.001
BMI (mean (SD))	22.42 (3.28)	22.21 (3.49)	0.662
First intercourse (age) (mean (SD))	16.68 (2.04)	16.21 (1.83)	0.095
Antibiotics (last 2 weeks) = Yes (n (%))	5 (5.2)	7 (7.6)	0.694
Menses (last 2 weeks) = Yes (n ((%))	55 (56.7)	44 (47.8)	0.282
Smoking (n ((%))			0.426
No	62 (63.9)	59 (64.1)	
Occasionally	16 (16.5)	10 (10.9)	
Regularly	19 (19.6)	23 (25.0)	
Lubricant use (last 2 weeks) = Yes (n ((%))	17 (17.5)	13 (14.1)	0.660
Intercourse with regular partner (last 2 weeks) = Yes (n ((%))	63 (64.9)	47 (51.1)	0.074
Intercourse with occasional partner (last 2 weeks) = Yes (n ((%))	11 (11.3)	14 (15.2)	0.568
Stress level (last 2 weeks) (n ((%))			0.548
0 (Min)	18 (18.6)	15 (16.3)	
1	42 (43.3)	37 (40.2)	
2	25 (25.8)	32 (34.8)	
3 (Max)	12 (12.4)	8 (8.7)	

A.2 Flow cytometry clustering analyses

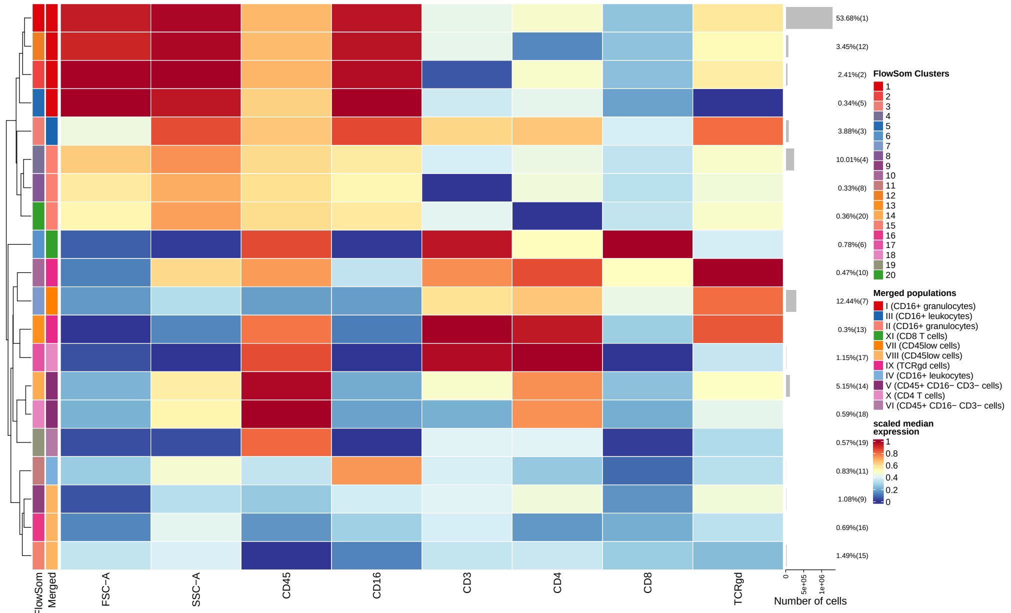

Fig A. Heatmap of 20 FlowSOM metaclusters and their annotation in 11 immune cells populations. Twenty FlowSOM metaclusters are identified on the left. Their merging and annotation into 11 immune cell populations is indicated in the second column named 'Merged'. Fluorescence is scaled on a per-marker basis. The total number of cells is shown in grey bar charts. The scale for the heatmap, which shows fluorescence intensity, is shown on the right. The code to generate this figure can be found in `https://doi.org/10.57745/KJGOYZ`.

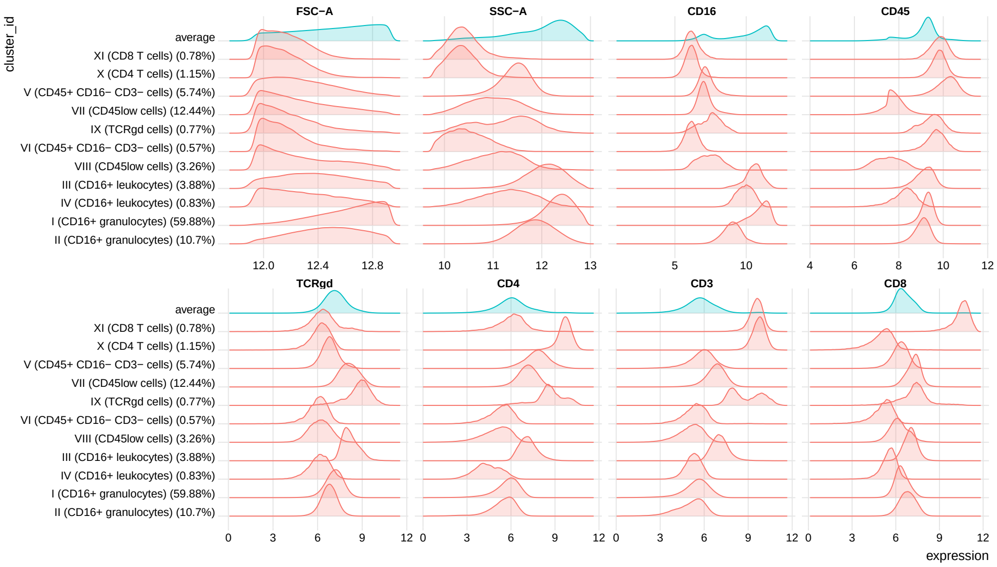

Fig B. Fluorescence intensity and annotation of the 11 immune cell populations identified. The distribution of the scaled intensities of FSC-A, SSC-A, and six fluorescent markers are individually represented for each of the 11 cell populations (one by row). The code to generate this figure can be found in `https://doi.org/10.57745/KJGOYZ`.

Table B. Immune cell clusters identification and annotation. 'Markers' indicates the fluorescence intensity of the key flow cytometry labels used to annotate each merged cluster and 'Commentary' indicates possible alternatives or characteristics of each cluster.

Cluster	Markers	Identification	Commentary
I	FSC^{high} SSC^{high} $CD45^{int}$ $CD3^{-}$ $CD4^{-}$ $CD16^{+}$ $TCR\gamma\delta^{-}$	FSC^{high} SSC^{high} $CD16^{high}$ cells	Probably majority of neutrophils
II	FSC^{int} SSC^{int} $CD16^{+}$ cells	FSC^{int} SSC^{int} $CD16^{+}$ cells	Possible NK cells, macrophages or Langerhans
III	FSC^{int} SSC^{hi} $CD45^{int}$ $CD3^{lo}$ $CD4^{lo}$ $CD16^{-}$ $TCR\gamma\delta^{int}$	$CD16^{+}$ $TCR\gamma\delta$ cells	
IV	FSC^{lo} SSC^{int} $CD45^{int}$ $CD3^{-}$ $CD4^{-}$ $CD16^{int}$ $TCR\gamma\delta^{-}$	$CD45^{low}$ $CD16^{+}$ leukocytes	Possible NK cells
V	FSC^{lo} SSC^{int} $CD45^{+}$ $CD3^{-}$ $CD4^{int}$ $CD16^{-}$ $TCR\gamma\delta^{-}$	$CD45^{+}$ $CD4^{int}$ leukocytes	Possible Monocytes, Macrophages or Langerhans cells
VI	FSC^{lo} SSC^{lo} $CD45^{+}$ $CD3^{-}$ $CD4^{-}$ $CD16^{-}$ $TCR\gamma\delta^{-}$	$CD45^{+}$ $CD3^{-}$ $CD16^{-}$ leukocytes	Possible B cells or ILCs
VII	FSC^{lo} SSC^{int} $CD45^{lo}$ $CD3^{lo}$ $CD4^{lo}$ $CD16^{-}$ $TCR\gamma\delta^{int}$	$CD45^{low}$ $TCR\gamma\delta$ cells	
VIII	FSC^{lo} SSC^{int} $CD45^{lo}$ $CD3^{-}$ $CD4^{-}$ $CD16^{-}$ $TCR\gamma\delta^{-}$	$CD45^{low}$ $CD3^{-}$ $CD16^{-}$ cells	Possible B cells, ILCs or progenitors
IX	FSC^{lo} SSC^{lo} $CD45^{+}$ $CD3^{int}$ $CD4^{+}$ $CD16^{-}$ $TCR\gamma\delta^{+}$	$CD45^{+}$ $TCR\gamma\delta$ cells	
X	FSC^{lo} SSC^{lo} $CD45^{+}$ $CD3^{+}$ $CD4^{+}$	CD4 T cells	
XI	FSC^{lo} SSC^{lo} $CD45^{+}$ $CD3^{+}$ $CD8^{+}$	CD8 T cells	Possibly includes MAIT cells

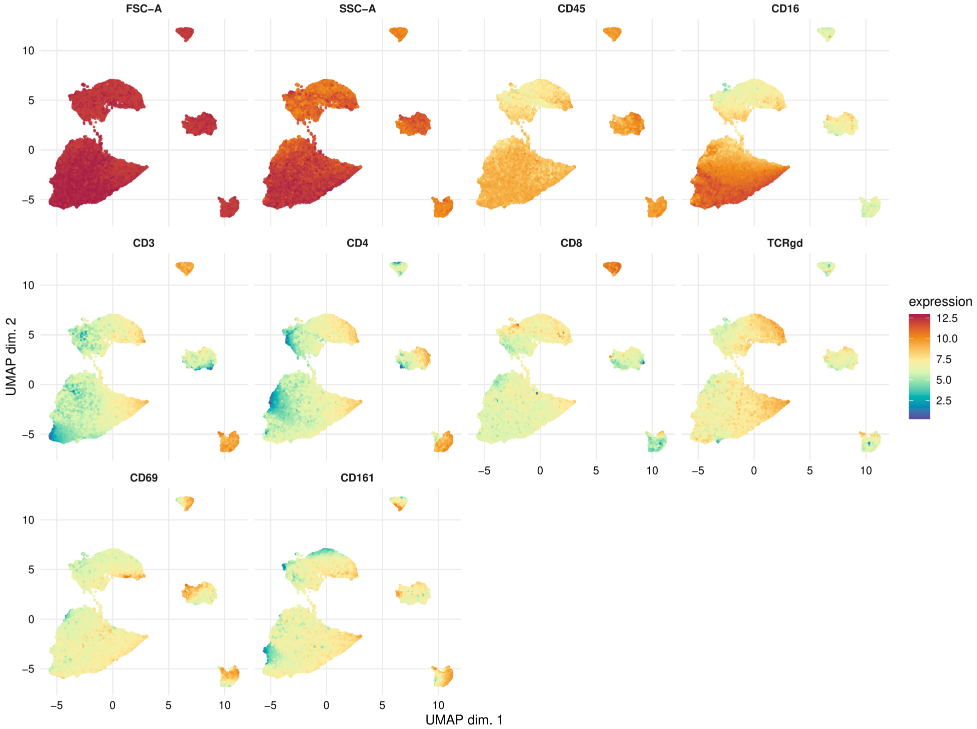

Fig C. Individual UMAPs per flow cytometry fluorescent label used in the FlowSom clustering. Each panel shows the scaled intensity for each fluorescent marker on the global UMAP displayed in Fig 1A. The code to generate this figure can be found in `https://doi.org/10.57745/KJGOYZ`.

Table C. Raw differential abundance results of the HPV focal infections *vs.* HPV negative samples. Full results of the differential abundance analysis shown in Fig 1B and 1C. Results were generated with the `diffcyt-DA-edgeR` function from the `diffcyt` package. 'logFC': *log* Fold Change of the cell population in samples positive for an HPV focal infections *vs.* HPV-negative samples. 'logCPM': *log* counts-per-million cells. 'LR': likelihood ratio statistics. 'p_val': p-value for the probability that the cell population is differently distributed in the two studied populations. 'p_adj': p-values adjusted for multiple testing by Benjamini-Bochberg correction.

	cluster_id	logFC	logCPM	LR	p_val	p_adj
1	I (CD16+ granulocytes)	-0.1508	18.4843	0.7362	0.3909	0.4493
2	II (CD16+ granulocytes)	-0.1345	16.4522	0.6954	0.4043	0.4493
3	III (CD16+ leukocytes)	-0.4086	15.8095	4.9753	0.0257	0.0367
4	IV (CD16+ leukocytes)	-0.6491	13.5607	10.6195	0.0011	0.0056
5	V (CD45+ CD16- CD3- cells)	-0.4345	16.0089	6.4819	0.0109	0.0272
6	VI (CD45+ CD16- CD3- cells)	-0.5672	14.2079	5.8560	0.0155	0.0310
7	VII (CD45low cells)	0.7614	17.5236	13.4581	0.0002	0.0024
8	VIII (CD45low cells)	0.4666	16.5201	5.1776	0.0229	0.0367
9	IX (TCRγδ cells)					
10	X (CD4 T cells)	-0.6797	15.6907	8.1342	0.0043	0.0145
11	XI (CD8 T cells)	-0.0262	15.3272	0.0098	0.9213	0.9213

Table D. Raw differential abundance results of the samples originating from vaccinated *vs.* non-vaccinated women. 'logFC' indicates the *log* Fold Change of the cell population in the 'vaccinated against HPV' *vs.* 'non-vaccinated against HPV' samples. See Fig C for details.

	cluster_id	logFC	logCPM	LR	p_val	p_adj
1	I (CD16+ granulocytes)	0.0062	18.4843	0.0012	0.9718	0.9718
2	II (CD16+ granulocytes)	0.1995	16.4522	1.5453	0.2138	0.5346
3	III (CD16+ leukocytes)	-0.2417	15.8095	1.7331	0.1880	0.5346
4	IV (CD16+ leukocytes)	0.0994	13.5607	0.2471	0.6191	0.7522
5	V (CD45+ CD16- CD3- cells)	0.5296	16.0089	9.6767	0.0019	0.0187
6	VI (CD45+ CD16- CD3- cells)	0.4047	14.2079	3.0140	0.0825	0.4127
7	VII (CD45low cells)	-0.2026	17.5236	0.9886	0.3201	0.6402
8	VIII (CD45low cells)	-0.0844	16.5201	0.1736	0.6769	0.7522
9	IX (TCRγδ cells)					
10	X (CD4 T cells)	0.1338	15.6907	0.3148	0.5748	0.7522
11	XI (CD8 T cells)	-0.1554	15.3272	0.3440	0.5575	0.7522

Table E. Differential expression analysis. Fold change (FC) value of focal HPV-positive over HPV negative samples and adjusted p-value for CD69 and CD161 fluorescence intensity for each of the 11 immune cell populations identified in flow cytometry analyses. Fold changes were calculated by an abundance analysis performed with `diffcyt-DS-limma` and adjusted with a Benjamini-Hochberg test.

Cluster	Marker	FC	p_val	p_adj
I	CD69	1.1610	0.0000	0.0000
II	CD69	1.1504	0.0000	0.0000
III	CD69	1.1916	0.0000	0.0000
IV	CD69	1.0820	0.0227	0.0340
V	CD69	1.2996	0.0000	0.0000
VI	CD69	0.9396	0.2407	0.2941
VII	CD69	1.0317	0.2887	0.3285
VIII	CD69	1.2023	0.0000	0.0000
IX	CD69	1.3375	0.0000	0.0000
X	CD69	0.8991	0.0278	0.0383
XI	CD69	1.0607	0.3458	0.3804
I	CD161	1.2788	0.0000	0.0000
II	CD161	1.2544	0.0000	0.0000
III	CD161	1.1602	0.0000	0.0000
IV	CD161	1.3329	0.0000	0.0000
V	CD161	1.3559	0.0000	0.0000
VII	CD161	1.0985	0.0032	0.0050
VI	CD161	1.0962	0.0260	0.0373
VIII	CD161	0.7944	0.0005	0.0008
IX	CD161	1.5443	0.0000	0.0000
X	CD161	0.9648	0.4044	0.4305
XI	CD161	0.9079	0.0618	0.0816

A.3 Local cytokine concentrations

Table F. Summary of multivariate Bayesian model shown in Fig 2A. Columns indicate the name of the response variable, its nature (fixed or random effect), the estimated posterior mean, its standard error and, respectively, the 5% and 95% quantiles (quant.low and quant.high). As explained in the Methods, the five cytokines or chemokines were considered as separate response variables in a multivariate model built with the `mvbind` function from the `brms` R package.

response	effect	posterior mean	std.error	quant.low	quant.high
**Intercept**					
IFN-γ	fixed	0.93	0.06	0.81	1.06
IL-17A	fixed	0.93	0.08	0.77	1.08
CXCL10	fixed	2.93	0.09	2.74	3.11
CCL20	fixed	2.06	0.08	1.90	2.23
IL-1α	fixed	3.16	0.06	3.04	3.28
**HPV**					
IFN-γ	fixed	0.01	0.06	-0.11	0.13
IL-17A	fixed	-0.08	0.08	-0.23	0.07
CXCL10	fixed	-0.02	0.09	-0.20	0.16
CCL20	fixed	0.07	0.09	-0.11	0.25
IL-1α	fixed	-0.02	0.06	-0.14	0.11
**Standard deviation of the random effect per participant**					
IFN-γ	ran_pars	0.48	0.04	0.41	0.56
IL-17A	ran_pars	0.62	0.05	0.53	0.72
CXCL10	ran_pars	0.73	0.06	0.62	0.86
CCL20	ran_pars	0.55	0.06	0.43	0.67
IL-1α	ran_pars	0.44	0.04	0.36	0.52
**Pairwise correlations between variables**					
(IFN-γ, IL-17A)	ran_pars	0.61	0.07	0.45	0.74
(IFN-γ, CXCL10)	ran_pars	-0.26	0.11	-0.46	-0.04
(IL-17A, CXCL10)	ran_pars	0.10	0.11	-0.12	0.31
(IFN-γ, CCL20)	ran_pars	0.08	0.13	-0.17	0.34
(IL-17A, CCL20)	ran_pars	0.38	0.11	0.15	0.57
(CXCL10, CCL20)	ran_pars	0.51	0.10	0.30	0.68
(IFN-γ, IL-1α)	ran_pars	-0.11	0.12	-0.34	0.12
(IL-17A, IL-1α)	ran_pars	0.17	0.11	-0.05	0.38
(CXCL10, IL-1α)	ran_pars	-0.12	0.12	-0.35	0.12
(CCL20, IL-1α)	ran_pars	0.09	0.13	-0.17	0.34
Variable specific standard deviation					
IFN-γ	ran_pars	0.47	0.02	0.44	0.51
IL-17A	ran_pars	0.55	0.02	0.51	0.60
CXCL10	ran_pars	0.65	0.03	0.61	0.71
CCL20	ran_pars	0.71	0.03	0.66	0.77
IL-1α	ran_pars	0.46	0.02	0.43	0.49

Table G. R^2 for the Bayesian model shown in Fig 2A. The R^2 for the brms model in Fig 2A was calculated with the `loo_R2` function from the brms package using LOO-residuals and Bayesian bootstrap.

	Estimate	Est.Error	Q2.5	Q97.5
R^2_IFNγ	0.49	0.05	0.38	0.57
R^2_IL-17A	0.54	0.04	0.46	0.61
R^2_CXCL10	0.46	0.05	0.36	0.56
R^2_CCL20	0.20	0.03	0.13	0.26
R^2_IL-1α	0.40	0.06	0.27	0.50

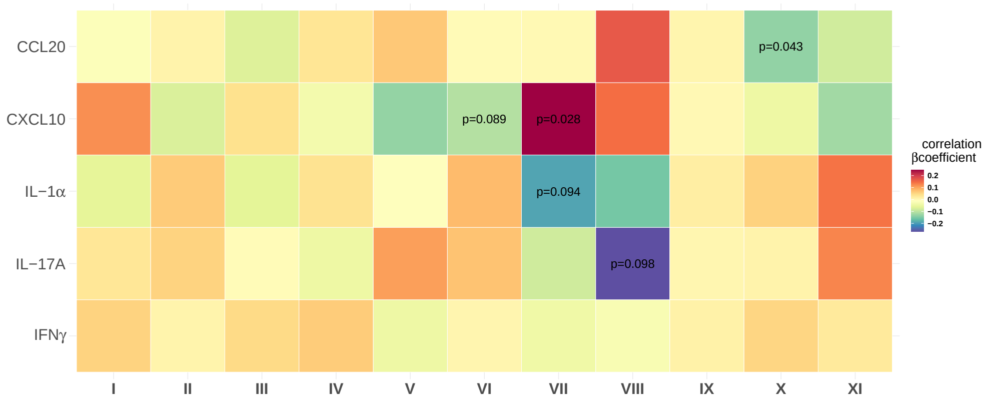

Fig D. Local immune response in HPV negative samples. Correlation matrix between the local density of five cytokines and the proportion of the 11 cell clusters from Fig 2. β represents the regression coefficient of linear regression with each cell population as the response variable (see Methods). For instance, a β of 0.14 can be interpreted as 'a 10 percentage point increase in the CD8 T cells frequency is associated with a 1.4 percentage point increase in the IFNγ concentration'. The code to generate this figure can be found in `https://doi.org/10.57745/KJGOYZ`.

A.4 Viral kinetics

A.4.1 Individual infection duration

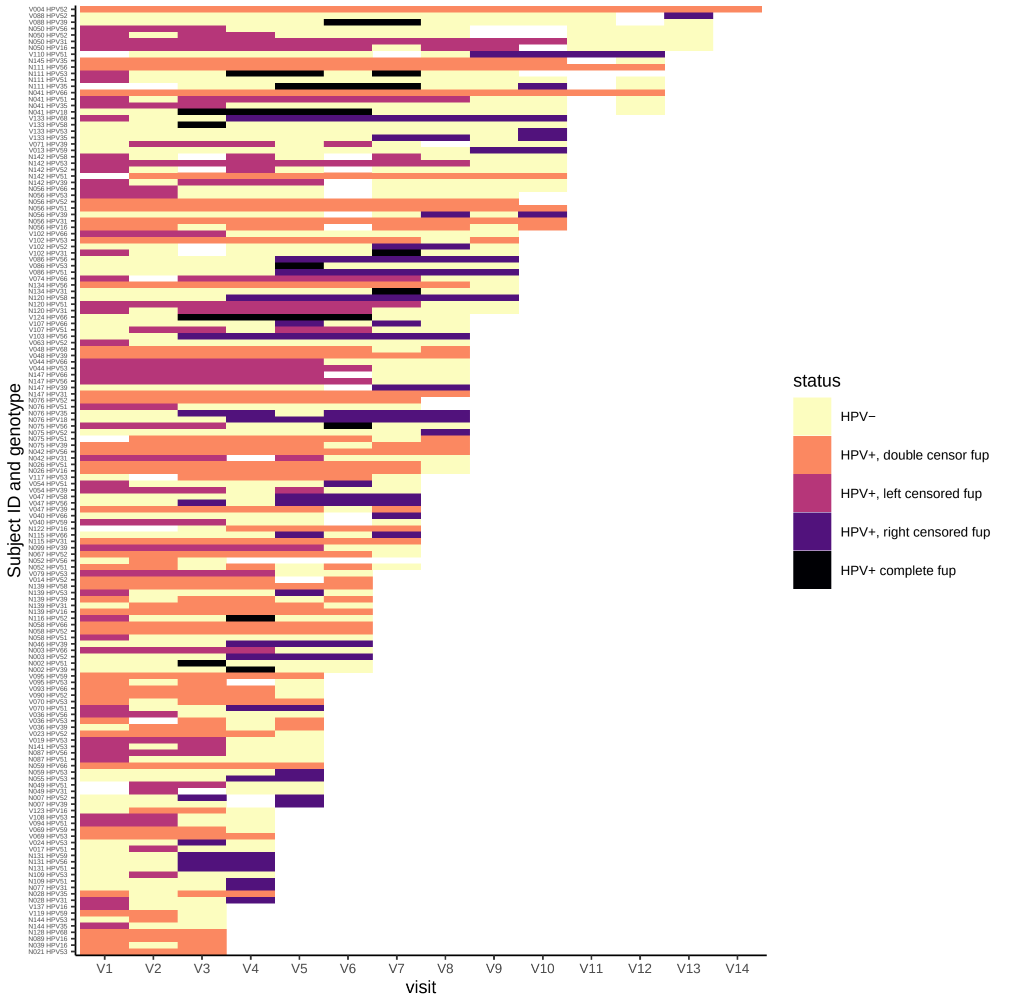

Fig E. HPV infections stratified by censoring status. The figure indicates the qPCR HPV status for each participant visit in the PAPLCEAR cohort. The color indicates whether the sample was positive for a given HPV genotype, and if so, if the follow-up was censored, *i.e.* we missed the beginning or the clearance of the infection. White cells indicate missing information, due to low sample quality (less than 100 albumin copies). The code to generate this figure can be found in `https://doi.org/10.57745/KJGOYZ`.

A.4.2 Parameters estimates

Table H. Parameters estimates for the viral kinetics three-slope model. We summarise the posterior distribution of each parameter, which is an aggregation of the 3,000 samplings on 4 MCMC chains, with its mean value, standard deviation, standard error of the mean. The distribution is then characterized by the 2.5^{th}, 50th and 97.5^{th} quantiles. N_{eff} is the effective sample size, correcting for the potential autocorrelation between the samples within a chain, and $\hat{R}$ is a metric indicating if the chains have converged (and then tends to 1).

	mean	se_mean	sd	2.5%	25 %	50 %	75 %	97.5%	n_eff	Rhat
$\log(\psi_{\text{gr}})$	-0.45	0.01	0.36	-1.23	-0.67	-0.42	-0.19	0.16	3560.23	1.00
$\log(\mu_{\text{p}})$	2.69	0.00	0.15	2.40	2.59	2.69	2.78	2.98	1764.86	1.00
$\log(\psi_{\text{cl}})$	-0.11	0.01	0.26	-0.66	-0.27	-0.09	0.07	0.34	2213.84	1.01
$\log(\mu_{\text{vl}})$	1.47	0.00	0.08	1.30	1.42	1.47	1.53	1.63	3343.95	1.00
ω_{η_p}	0.65	0.00	0.06	0.54	0.61	0.65	0.69	0.77	870.06	1.00
$\omega_{\eta_{\text{vl}}}$	0.21	0.00	0.03	0.15	0.19	0.21	0.23	0.28	370.96	1.02
ω_{ρ_p}	0.39	0.00	0.05	0.29	0.35	0.38	0.42	0.49	378.71	1.01
$\omega_{\rho_{\text{vl}}}$	0.28	0.00	0.07	0.18	0.23	0.27	0.32	0.46	4355.92	1.00
$L_\rho[2,1]$	0.04	0.00	0.20	-0.36	-0.10	0.04	0.18	0.43	2911.13	1.00
$L_\rho[2,2]$	0.98	0.00	0.03	0.89	0.97	0.99	1.00	1.00	4934.35	1.00
$L_\eta[2,1]$	0.33	0.03	0.15	0.02	0.22	0.33	0.44	0.61	27.66	1.07
$L_\eta[2,2]$	0.93	0.01	0.06	0.79	0.90	0.94	0.98	1.00	43.62	1.05
ν_p	-2.07	0.01	0.51	-3.10	-2.39	-2.07	-1.76	-1.06	6176.66	1.00
ν_{vl}	-0.58	0.00	0.27	-1.20	-0.72	-0.55	-0.40	-0.15	5415.05	1.00
σ_{vl}	1.33	0.00	0.04	1.26	1.31	1.33	1.36	1.41	495.28	1.01

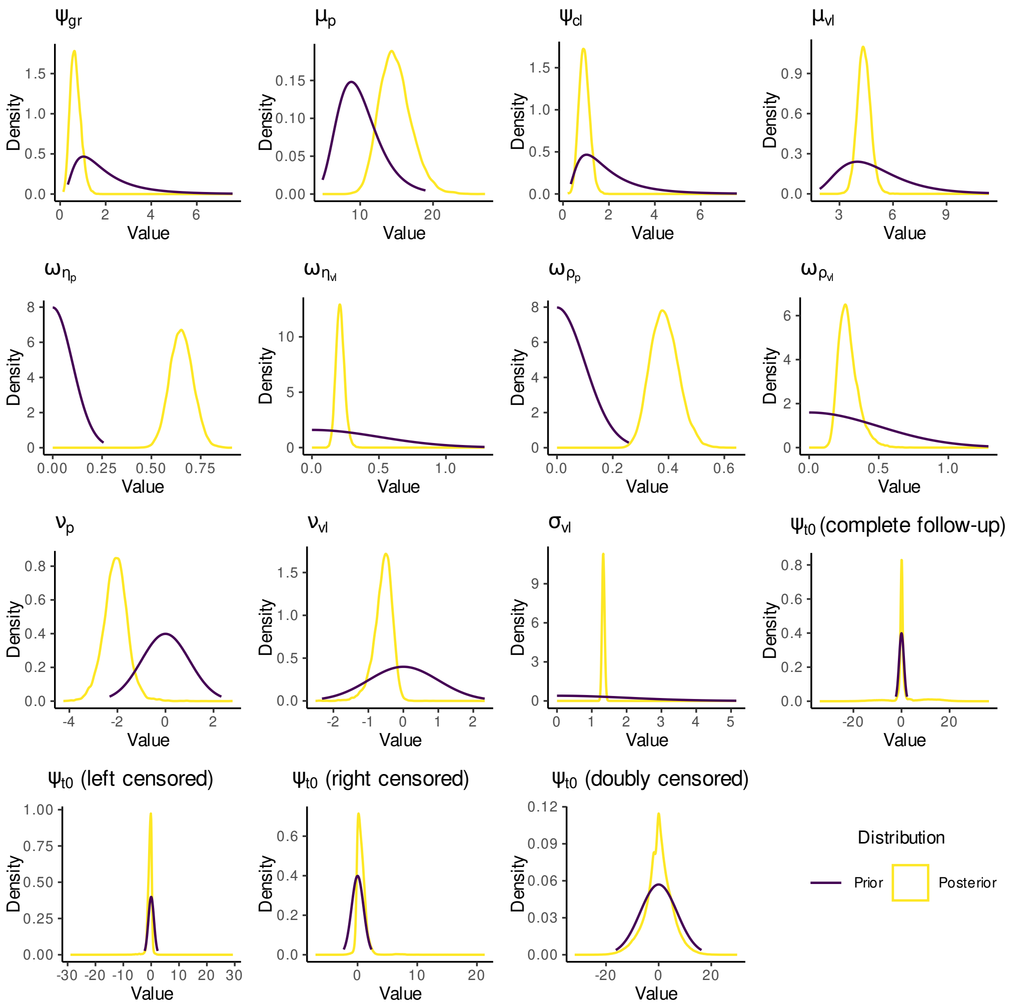

Fig F. Prior and posterior distribution of each parameter governing the viral kinetics. The code to generate this figure can be found in `https://doi.org/10.57745/KJGOYZ`.

A.4.3 HPV genotypes random effects

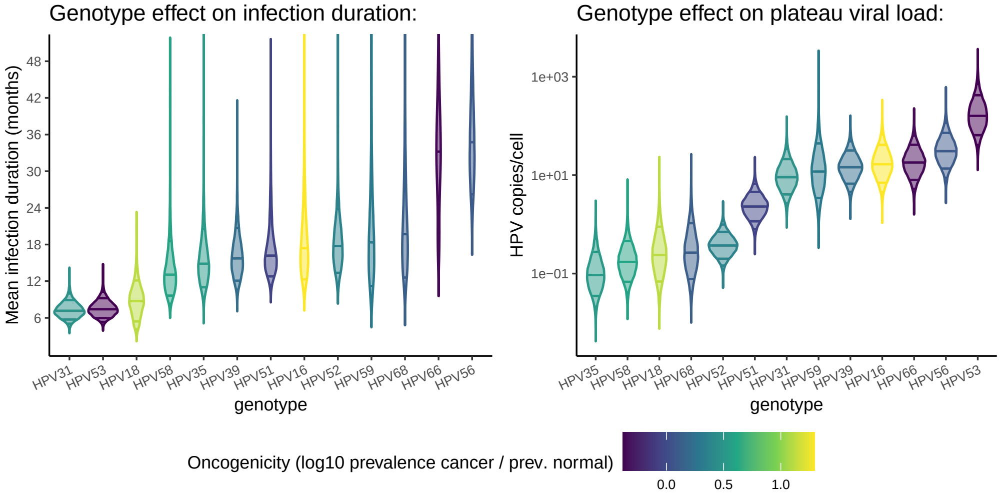

Fig G. Posterior distribution of random effects associated with the genotype. The horizontal lines in the violins show the 2.5^{th}, 25th, 50th, 75th and 97.5^{th} percentiles of the posterior distribution. The color shows the HPV genotype oncogenicity, which was estimated through the log10 of the ratio between its prevalence in invasive cervical cancers and in normal cytologies (data `hpvcentre.net`). The code to generate this figure can be found in `https://doi.org/10.57745/KJGOYZ`.

A.4.4 Sensitivity analyses

We performed sensitivity analyses to assess the importance of three assumptions we had to make.

1. We had to restrict the hyperprior distribution (i.e. the prior for ω_η and ω_ρ) in order to obtain a realistic distribution of infection duration of the doubly censored follow-ups. We assessed the sensitivity of the choice of the prior's standard deviation (sd) by increasing it from its default value, 0.1, to 0.2 and 0.5.
2. Instead of considering that an infection was cleared if there were two successive negative visits, we extended the threshold to 3 successive negative measurements, which reduced the number of re-infections from 10 to 3.
3. We considered a concurrent virus kinetics model without the plateau phase, *i.e.* only with an exponential increase phase and an exponential decrease phase.

Fig H shows how varying the hyperprior standard deviation affects the posterior distribution of the cumulative distribution of the infection duration. When the follow-ups are doubly censored (red curve), the hyperprior choice had a strong impact on the distribution of the infection duration.

Fig I shows the effect of the number of successive negative visits to define infection clearance on the infection duration distribution. This had little impact on the overall distribution.

Fig J shows a posterior predictive check performed for a model with or without a plateau phase. Centered on the time from the empirical infection midpoint, similarly to our model, we compare the distribution of the viral load quartiles through time. For the model without the plateau phase, the data simulated from the posterior distribution has difficulties in capturing the empirical virus load values of the 75th percentile, *i.e* the infections lasting the longest or having the highest viral load.

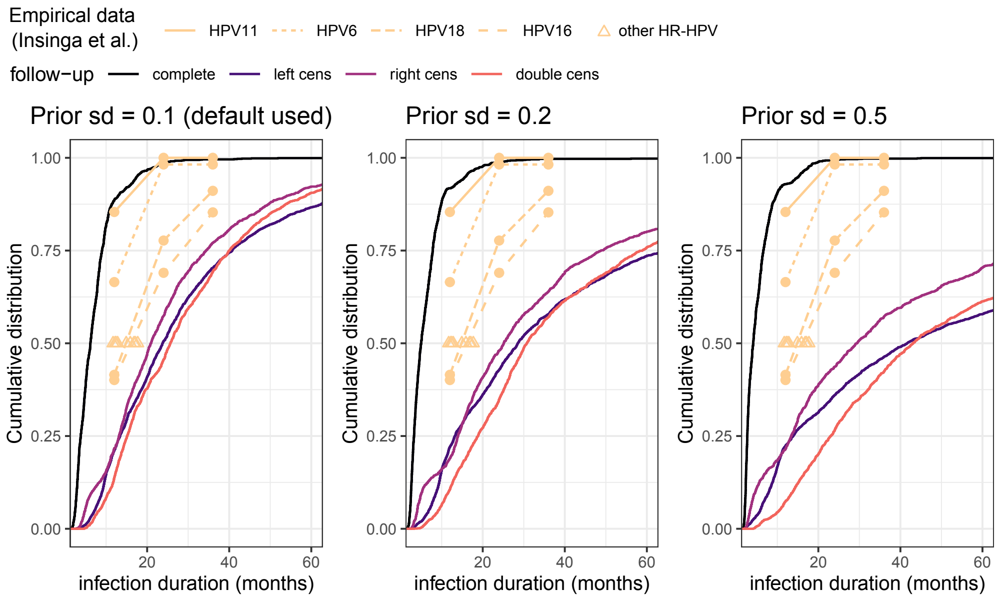

Fig H. Sensitivity analysis of the choice of hyperprior on the random effects governing the infection duration. We plot the empirical cumulative distribution of infections simulated from the posterior distribution, as a function of the follow-up censoring. The empirical distributions estimated by [S1] are shown for comparison. We compare three choices of priors standard deviation of the half-normal distribution governing the parameters ω_η and ω_ρ. The code to generate this figure can be found in `https://doi.org/10.57745/KJGOYZ`.

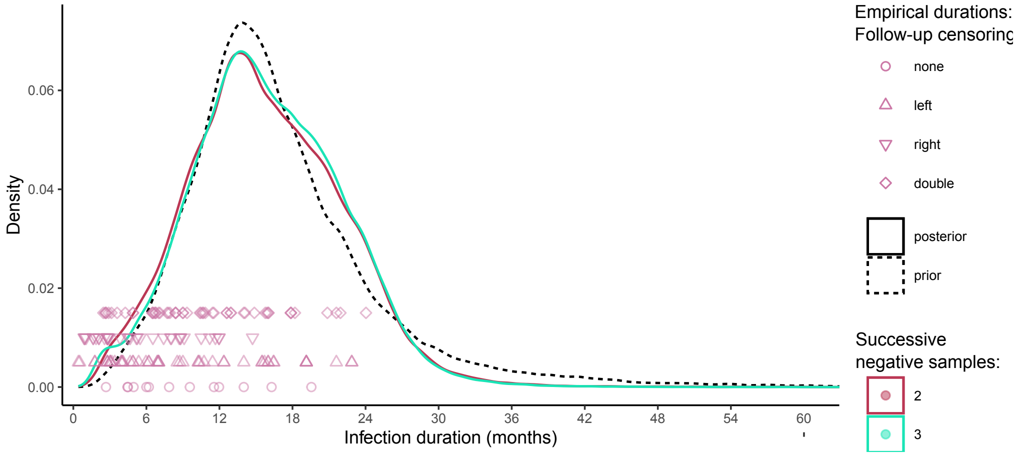

Fig I. Prior and posterior distribution of the duration of the infections compared to the observed durations, as a function of the number of successive negative samples chosen to define a new infection. The code to generate this figure can be found in `https://doi.org/10.57745/KJGOYZ`.

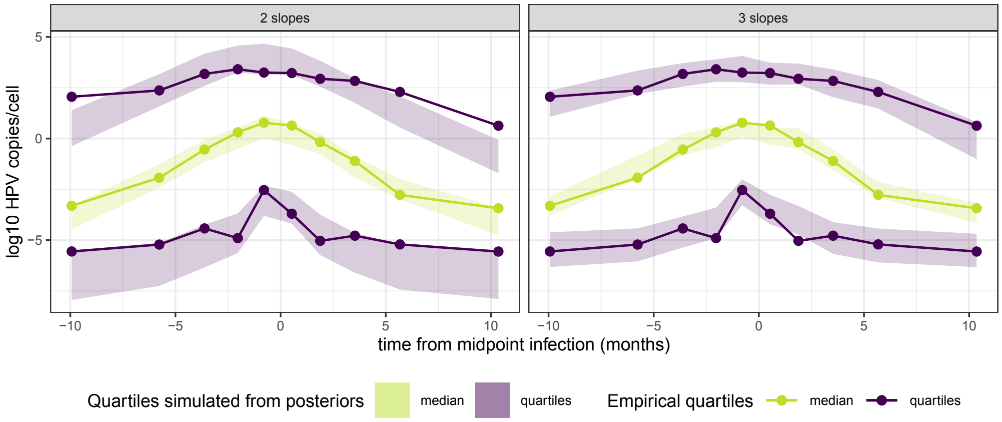

Fig J. Posterior predictive check of the viral kinetics comparing a model with two slopes and a model with a plateau. Aligning all follow-ups to the time of their midpoint infection, we split the times into twelve bins with evenly distributed observations. We compare the viral load quartiles (25th, 50th and 75th percentiles) in those time bins (plain lines), to the quartiles obtained from the simulations. We show the uncertainty by representing the 90% CrI of these quartiles, when assuming a model with an exponential increase and decrease of the viral load (2 slopes) or a model including a plateau (3 slopes). The code to generate this figure can be found in `https://doi.org/10.57745/KJGOYZ`.

A.5 Immune response kinetics modelling

A.5.1 Parameters estimates

parameter	immune variable	mean	sd	2.5%	50%	97.5%
μ_{t0}	IFNγ	1.01	0.10	0.80	1.00	1.18
μ_{tm}	IFNγ	-0.15	0.22	-0.58	-0.14	0.25
μ_{delay}	IFNγ	0.40	0.19	0.09	0.38	0.78
μ_{t0}	IL 17A	0.83	0.11	0.60	0.83	1.04
μ_{tm}	IL 17A	0.00	0.22	-0.45	0.01	0.47
μ_{delay}	IL 17A	0.31	0.21	0.05	0.26	0.81
μ_{t0}	CXCL10	2.77	0.11	2.57	2.77	2.97
μ_{tm}	CXCL10	-0.00	0.15	-0.28	-0.00	0.33
μ_{delay}	CXCL10	0.48	0.16	0.18	0.49	0.79
μ_{t0}	CCL20	1.94	0.10	1.75	1.94	2.13
μ_{tm}	CCL20	0.17	0.19	-0.20	0.15	0.55
μ_{delay}	CCL20	0.38	0.18	0.11	0.36	0.77
μ_{t0}	IL1α	3.13	0.08	2.98	3.14	3.28
μ_{tm}	IL1α	0.01	0.12	-0.24	0.01	0.27
μ_{delay}	IL1α	0.49	0.19	0.16	0.49	0.84

Table I. Fixed effects estimates for the cytokines two-slopes model. We summarise the posterior distribution of each parameter, aggregated from the 500 bootstrapped models. We therefore could not provide any convergence statistics per run.

Tue Feb 6 10:20:15 2024

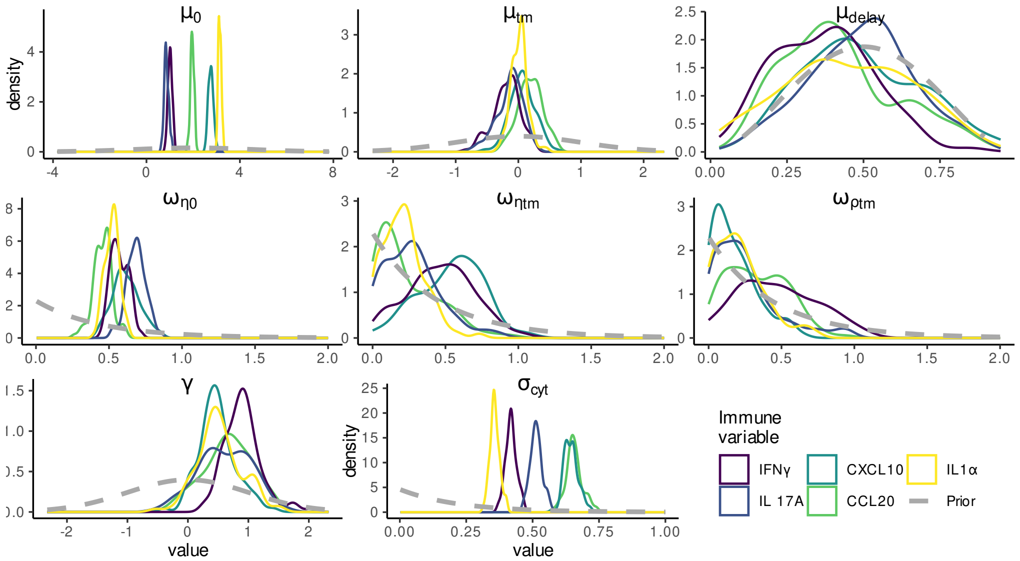

Fig K. Prior and posterior distribution of each parameter governing the cytokine response kinetics. Each color represents the distribution for one cytokine, and the dotted grey line represents the prior. The interval represents 99% of the prior distribution. The code to generate this figure can be found in `https://doi.org/10.57745/KJGOYZ`.

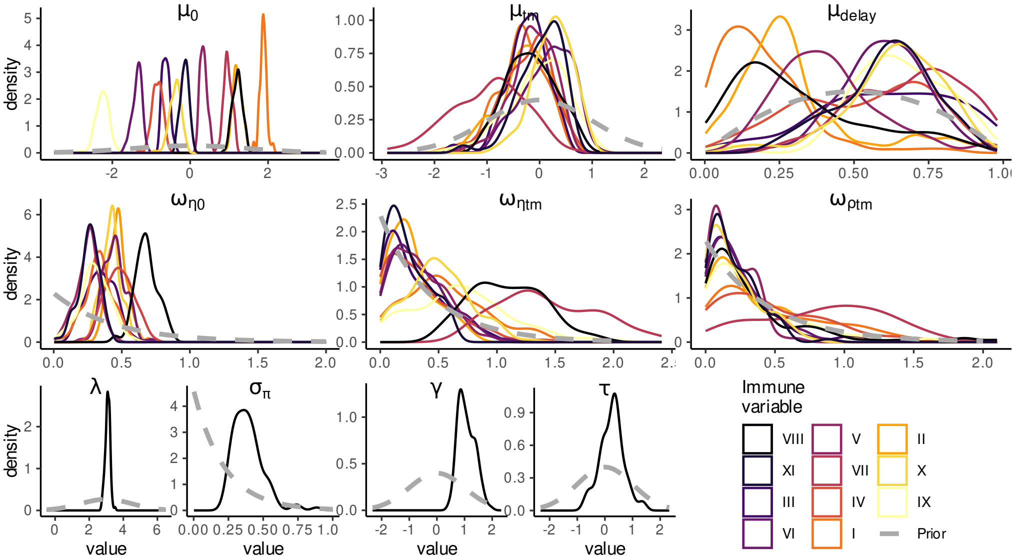

Fig L. Prior and posterior distribution of each parameter governing the leukocytes response kinetics. Each color represents the distribution for one leukocyte population, and the dotted grey line represents the prior. On the bottom row, the parameters are identical for all leukocyte populations, and the black line represents the prior. The interval represents 99% of the prior distribution. The code to generate this figure can be found in `https://doi.org/10.57745/KJGOYZ`.

parameter	immune variable	mean	sd	2.5%	50%	97.5%
μ_{delay}	I	0.23	0.15	0.05	0.20	0.68
μ_{tm}	I	0.39	0.27	-0.24	0.41	0.85
μ_{t_0}	I	1.90	0.12	1.67	1.89	2.12
μ_{delay}	II	0.23	0.18	0.04	0.20	0.83
μ_{tm}	II	0.20	0.32	-0.48	0.22	0.69
μ_{t_0}	II	1.29	0.12	1.06	1.29	1.53
μ_{delay}	III	0.45	0.20	0.12	0.44	0.89
μ_{tm}	III	-0.22	0.48	-1.43	-0.13	0.52
μ_{t_0}	III	-0.38	0.11	-0.59	-0.38	-0.18
μ_{delay}	IV	0.45	0.21	0.13	0.41	0.88
μ_{tm}	IV	0.18	0.36	-0.64	0.21	0.76
μ_{t_0}	IV	-0.80	0.14	-1.07	-0.80	-0.51
μ_{delay}	V	0.46	0.15	0.21	0.45	0.77
μ_{tm}	V	0.06	0.38	-0.80	0.11	0.64
μ_{t_0}	V	0.47	0.11	0.24	0.47	0.66
μ_{delay}	VI	0.63	0.13	0.38	0.63	0.85
μ_{tm}	VI	0.35	0.31	-0.40	0.39	0.84
μ_{t_0}	VI	-1.29	0.14	-1.57	-1.28	-0.99
μ_{delay}	VII	0.84	0.18	0.21	0.90	0.99
μ_{tm}	VII	0.21	0.42	-0.75	0.26	0.84
μ_{t_0}	VII	0.65	0.14	0.38	0.66	0.96
μ_{delay}	VIII	0.63	0.22	0.08	0.70	0.91
μ_{tm}	VIII	0.43	0.34	-0.35	0.46	1.02
μ_{t_0}	VIII	0.97	0.16	0.65	0.97	1.28
μ_{delay}	IX	0.70	0.15	0.30	0.71	0.92
μ_{tm}	IX	0.49	0.35	-0.39	0.54	1.02
μ_{t_0}	IX	-2.27	0.18	-2.67	-2.26	-1.94
μ_{delay}	X	0.70	0.13	0.39	0.71	0.90
μ_{tm}	X	0.44	0.33	-0.34	0.48	0.95
μ_{t_0}	X	-0.32	0.16	-0.65	-0.32	-0.03
μ_{delay}	XI	0.67	0.13	0.31	0.68	0.89
μ_{tm}	XI	0.44	0.28	-0.23	0.48	0.87
μ_{t_0}	XI	-0.23	0.13	-0.49	-0.23	0.02

Table J. Fixed effects estimates for the FCM two-slopes model. We summarise the posterior distribution of each parameter, aggregated from the 500 bootstrapped models. We therefore could not provide any convergence statistics per run.

A.5.2 Random effects correlation matrix

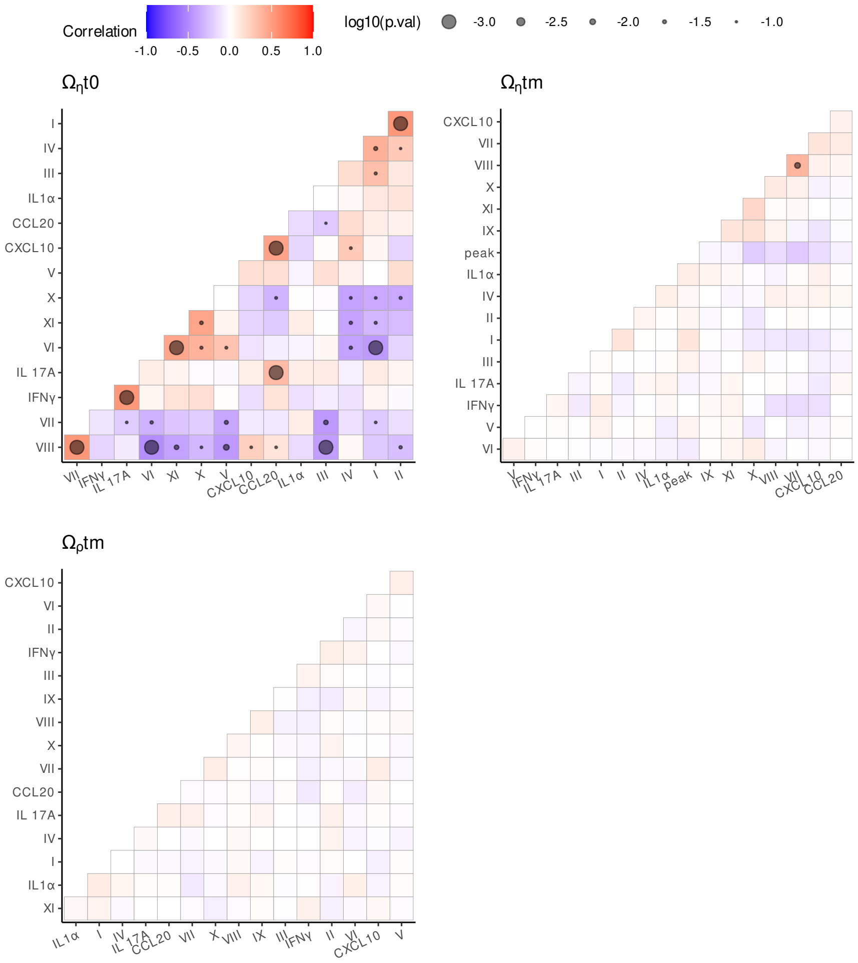

Fig M. Correlation between random effects of the different immune variables. Parameter ψ_0 is associated with a host-level covariance matrix $\Omega_{\eta 0}$ and a genotype-level covariance matrix $\Omega_{\rho 0}$. The parameters ψ_{tm} and ψ_{delay} are associated jointly with a host-level covariance matrix $\Omega_{\eta tm}$. The colour indicates the median posterior correlation between a pair of parameters and the dots indicate the proportion of the posterior samples not in the same sign as the median, in a log10 scale. To improve the clarity of the plot, only pairs for which the posterior correlation has a different sign in less than 10% of the samples are shown with a dot. The code to generate this figure can be found in `https://doi.org/10.57745/KJGOYZ`.

A.5.3 Immune response associated with the IgG serum titer

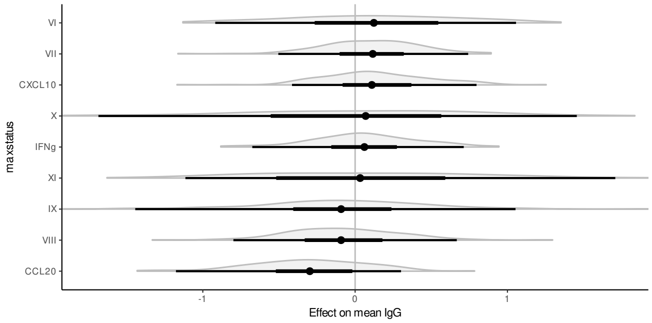

Fig N. Linear regression of the IgG mean serum titer as a function of the mean cells clusters frequencies and log cytokines concentration. We selected the cell clusters that appeared later in the infection *i.e.* associated with an adaptive immune response, and the cytokines that showed a potential response to the infection at the population level. The thin lines represent the 95% CrI, the thick lines the interquartile ranges, and the dot the median of the posterior distribution. The code to generate this figure can be found in `https://doi.org/10.57745/KJGOYZ`.

B Supplementary methods

B.1 Flow diagram of the sample selection

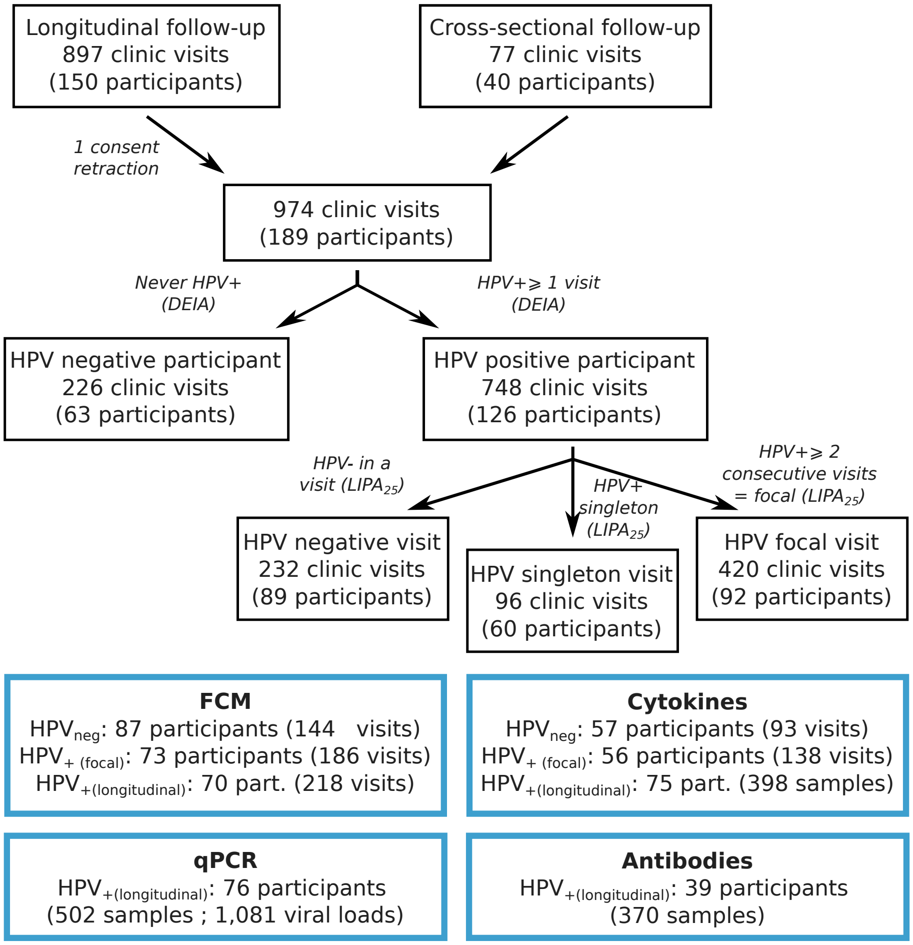

Fig O. Sample selection diagram. The bottom boxes indicate the number and nature of samples used in each type of analysis.

B.2 Parameters and priors used for the Bayesian models

B.2.1 Rstan settings

Table K. Rstan model settings.

Model	Warm-up	Chains	Adapt delta	Max treedepth	Sampling
Viral kinetics	3,000	4	0.99	14	3,000
Immune response	1,000	500 times 1	0.99	13	500
Serological status	1,000	4	0.99	10	1,000

B.2.2 Priors for the viral kinetics

Table L. Priors used for the viral kinetics parameters. LKJ indicates the Lewandowski, Kurowicka, and Joe distribution [S2]. In the first column, the vector length is indicated by the parentheses '()' and $L()$ denotes a lower-triangular matrix. For the normal distribution ($\mathcal{N}$), the two parameters are the mean and standard deviation (sd), and the"[]" represents the distribution truncation. For the LogNormal distribution, the parameters correspond to the mean and sd of the lognormally distributed quantity.

Param.	Short description	Unit	Prior distribution
ψ_{gr}	Viral load growth duration	months	$\text{LogNormal}(2, 1.5)$
ψ_{cl}	Viral load clearance duration	months	$\text{LogNormal}(2, 1.5)$
μ_p	Plateau duration	months	$\text{LogNormal}(10, 3)$
μ_{vl}	Plateau viral load	$\log_{10}$ HPV copies /cell	$\text{LogNormal}(5, 2)$
ψ_{t0}	Date of infection	months	dependent on the follow-up censoring (see methods)
v_0	initial viral load	$\log_{10}$ HPV copies /cell	set to 10^{-4}
$\omega_\eta(2)$	host-level random effects variance	(months, $\log_{10}$ HPV copies /cell$)^2$	$\mathcal{N}(0, (0.1, 0.5))[0,]$
$\omega_\rho(2)$	genotype-level random effects variance	(months, $\log_{10}$ HPV copies /cell$)^2$	$\mathcal{N}(0, (0.1, 0.5))[0,]$
L_η L(2,2)	Chol.-decomposed matrix for host-level random effects covariance		LKJ(1)
L_ρ L(2,2)	Chol.-decomposed matrix for genotype-level random effects covariance		LKJ(1)
ν_p	Reinfection mean effect on plateau duration	months	$\mathcal{N}(0, 1)$
ν_{vl}	Reinfection mean effect on plateau viral load	$\log_{10}$ HPV copies /cell	$\mathcal{N}(0, 1)$
σ_{vl}	residual error	$\log_{10}$ HPV copies /cell	$\mathcal{N}(0, 1)[0,]$

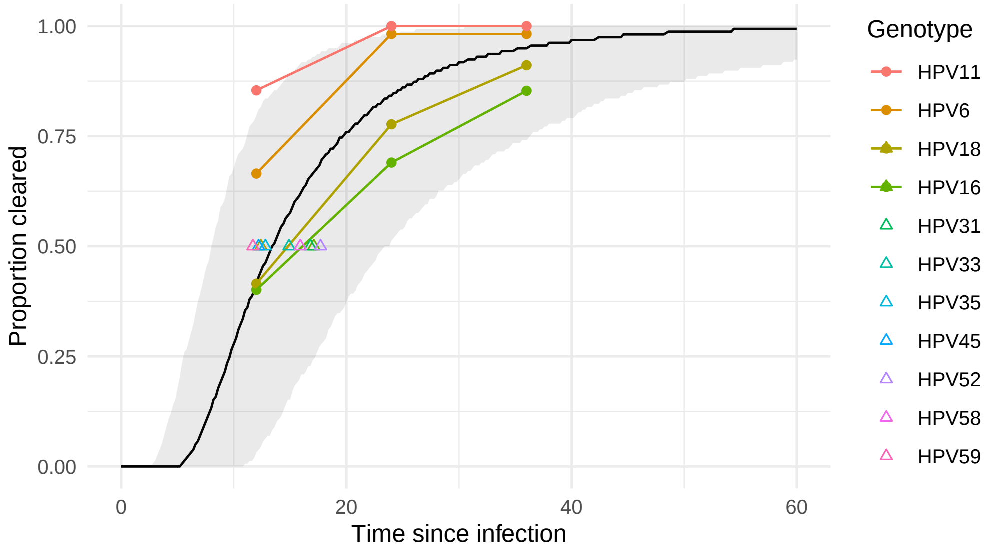

Fig P. Prior predictive check of the infection duration. We simulated 1,000 datasets with the same structure (number of participants and number of co-infections) as the PAPCLEAR cohort by sampling all the parameters from the prior distributions, except the random effects standard deviations ω_η and ω_ρ that were fixed. Following [S3], we used an intermediate version of prior predictive check by sampling the hyperpriors ω_η and ω_ρ in the posterior distribution. The black line indicates the median value, and the grey area, the 95% interval of the sampled infection duration. For reference, infection duration distributions from [S1] are shown for various genotypes. Note that wart-causing HPV6 and HPV11 are known to cause shorter infections than oncogenic types but were not followed in our study. The code to generate this figure can be found in `https://doi.org/10.57745/KJGOYZ`.

B.2.3 Priors for the immune response

The other parameters depended on the type of immune marker:

Table M. Priors used for the FCM dynamics parameters. 'clr' stands for center-log ratio transform. See Table L for details.

Param.	Short description	Unit	Prior distribution
$\mu_0(10)$	Baseline value relative to the baseline	clr frequency	$\mathcal{N}(0, 1.5)$
$\mu_{\text{tm}}(11)$	Peak value relative to the baseline	clr frequency	$\mathcal{N}(0, 1)$
$\mu_{\text{delay}}(11)$	Peak time	proportion of infection duration	$\text{Beta}(2, 2)$
λ	Assay precision mean value	Number of cells	$\mathcal{N}(\log(20), 1.5)$
σ_π	Assay precision std. dev	Number of cells	$\mathcal{E}(1)$
$\pi(11)$	Precision of the assay	Number of cells	$\mathcal{N}(\lambda, \sigma_\pi)$
γ_{FCM}	Coinfection effect reduction coefficient		$\mathcal{N}(0, 1)$

Table N. Priors used for the cytokine dynamics parameters. 'std' stands for standardized concentration, *i.e.* divided by the total protein concentration. See Table L for details.

Param.	Short description	Unit	Prior distribution
$\mu_0(5)$	Baseline value	log(std. concentration)	$\mathcal{N}(\text{mean } 2, \text{sd } 2.5)$
$mu_{\text{tm}}(5)$	Peak/Drop value	log(std. concentration)	$\mathcal{N}(\text{mean } 0, \text{sd } 1)$
$\mu_{\text{delay}}(5)$	Peak time	proportion of infection duration	$\text{Beta}(3, 3)$
$\sigma_{\text{cyt}}(5)$	Variance of the assay error	log(std. concentration)^2	$\mathcal{E}(-\log(0.01)/1)$
L_{cyt} L(5,5)	Chol.-decomposed matrix for error covariance		LKJ(1)
$\gamma(5)$	Coinfection effect reduction coefficient		$\mathcal{N}(0, 1)$

Table O. Priors used for both the random effects of both FCM and cytokines dynamics parameters, using a common variance-covariance matrix. See Table L for details. When a vector is of different units, we mention them in the order of the values.

Param.	Short description	Unit	Prior distrib.
$\omega_{\eta_0}(15)$	Baseline value individual-level random effect variance	(log(std. concentration), clr(frequency))^2	$\mathcal{E}(-\log(0.01)/2)$
$\omega_{\eta_{tm}}(16)$	Peak/drop value individual-level random effect variance	(log(std. concentration), clr(frequency))^2	$\mathcal{E}(-\log(0.01)/2)$
$\omega_{\eta_{\text{delay}}}(1)$	Peak timing individual-level random effect variance		$\mathcal{E}(-\log(0.01)/2)$
$\omega_{\rho_{tm}}(16)$	Peak/drop value genotype-level random effect variance	(log(std. concentration), clr(frequency))^2	$\mathcal{E}(-\log(0.01)/2)$
L_{η_0} $L(15,15)$	Chol.-decomposed matrix for genotype-level random effects covariance		LKJ(0.75)
$L_{\eta_{tm},\eta_{\text{delay}}}$ $L(17,17)$	Chol.-decomposed matrix for genotype-level random effects covariance		LKJ(0.75)
$L_{\rho_{tm}}$ $L(16,16)$	Chol.-decomposed matrix for genotype-level random effects covariance		LKJ(0.75)
τ	Regression parameter of peak time and infection duration		$\mathcal{N}(0.5, 0.5)$

C Authors' contribution

Nicolas Tessandier structured and analysed the databases, implemented the flow cytometry data, led immunological analyses, performed statistical analyses, and wrote the manuscript with input from all authors.

Baptiste Elie structured and analysed the databases, led the Bayesian hierarchical modelling, performed immunological analyses, and wrote the manuscript with input from all authors.

Christian Selinger structured and analysed the databases.

Claire Bernat implemented some of the laboratory protocols and processed part of the samples.

Vanina Boué implemented the qPCR protocols and processed some of the samples, including all the qPCR experiments.

Soraya Groc processed some of the samples.

Massilva Rahmoun provided immunological and flow cytometry expertise and coordinated the clinical study and the sample processing.

Bastien Reyné helped to structure and analyse the databases.

Anne-Sophie Bedin provided expertise for the flow cytometry protocol and analyses.

Thomas Beneteau provided statistical expertise.

Marine Bonneau performed some of the clinical visits and provided expertise.

Christelle Graf performed some of the clinical visits and provided expertise.

Jérémie Guedj provided critical statistical expertise.

Nathalie Jacobs provided critical immunological and flow cytometry expertise.

Tsukushi Kamiya provided critical statistical expertise.

Marion Kerioui provided critical statistical expertise.

Julie Lajoie provided critical immunological and flow cytometry expertise.

Imène Melki provided critical immunological and flow cytometry expertise.

Jean-Luc Prétet provided virology expertise to set up the qPCR.

Géraldine Schlecht-Louf provided critical immunological expertise and helped with the interpretation of the flow cytometry data.

Mircea T. Sofonea provided critical statistical expertise.

Olivier Supplisson provided critical statistical expertise.

Chris Wymant provided critical statistical expertise and feedback on the manuscript.

Tim Waterboer provided critical immunological expertise and his team performed the antibody titrations.

Christophe Hirtz provided immunological expertise and his team performed the cytokines dosages.

Vincent Foulongne provided virology expertise and his laboratory hosted most of the experiments.

Marie-Christine Picot provided epidemiological expertise and her team set up the case reporting form.

Jacques Reynes provided epidemiological expertise and was the lead clinical investigator in the study.

Vincent Tribout provided epidemiological expertise and his team of the STI detection centre performed the clinical visits.

Édouard Tuaillon provided immunological expertise and his laboratory provided the support for the flow cytometry analyses.

Ignacio G Bravo provided critical expertise on HPVs and in virology in general.

Michel Segondy was one of the co-PIs of the clinical study, provided virology expertise and his laboratory hosted most of the experiments.

Nathalie Boulle was one of the co-PIs of the clinical study, provided HPV expertise and her laboratory performed cervical lesion screening.

Carmen Lia Murall contributed to the conception of the project, the implementation of the clinical study, and the data analysis.

Samuel Alizon conceived the project, implemented the clinical study, structured and analysed the databases, and wrote the first version of the manuscript and the revised version with input from all authors.

Supplementary References

S1. Insinga RP et al. (2007) Incidence and duration of cervical human papillomavirus 6, 11, 16, and 18 infections in young women: an evaluation from multiple analytic perspectives. Cancer Epidemiol Biomarkers Prev 16: 709–15. DOI: 10.1158/1055-9965.EPI-06-0846.

S2. Lewandowski D, Kurowicka D, Joe H (2009) Generating random correlation matrices based on vines and extended onion method. Journal of Multivariate Analysis 100: 1989–2001. DOI: 10.1016/j.jmva.2009.04.008.

S3. Gelman A, Meng XL, Stern H (1996) Posterior predictive assessment of model fitness via realized discrepancies. Statistica sinica: 733–760.
