# Supplementary material for: Viral and immune dynamics of genital human papillomavirus infections in young women with high temporal resolution
Source: PLoS Biol. 2025 Jan 21;23(1):e3002949. doi: 10.1371/journal.pbio.3002949 (PMC11750104; doi:10.1371/journal.pbio.3002949)
Supplement: Attachment — Submitted filename: response_rev_plosbio.pdf [file pbio.3002949.s002.pdf]

Responses to the reviewers

Reviewer 1

Overview

In the submitted article, Viral and immune dynamics of human papillomavirus genital infections in young women, Tessandier and Elie et al. detail the establishment of the PAPCLEAR observational human cohort study enrolling HPV- and HPV+ women and utilized samples collected between 2016-2020 to study non-persistent HPV infections. From 189 participants, authors collected temporal viral titer (RT-PCR) and immune measure (clustered flow cytometry; cytokine; antibody) data, which were incorporated into a series of Bayesian models of viral dynamics and associated immune responses over time. The major findings of the study include i) identification of immune clusters from cervical smears that differ between HPV- and HPV+ participants; ii) generation of a three-slope HPV non-persistent infection model lasting approximately 14 months with a 12-month plateau bookended by rapid infection/clearance dynamics; iii) identified associations between CXCL10 and γδ T cells with viral clearance. This is a well-written manuscript, which does a thorough job explaining the modeling techniques used and establishes a novel cohort to answer critical questions related to common HPV infections. The clarity and manuscript would be improved with a few updates described below:

Reply: Thank you for this accurate summary and positive appreciation of our work!

Main Comments

Q 1.1 *Some of the model priors, such as infection duration and midpoint, are informed by the empirical data, but the rationale for hyperparameters of the decomposed terms is not fully explained. Since posterior parameter estimates are sensitive to the selection of priors, how would the authors quantify the robustness of the model (e.g. Sensitivity analysis)?*

Reply: We improved the explanations of the model but also seized the opportunity to revisit its structure, following suggestions from Reviewer #3. In the original version, we did not use the fact that we know whether or not an individual follow-up is truncated (this is why we estimated the midpoint of the infection). In the revised version, we treat the infection kinetics differently depending on whether or not we know the starting or ending date of the infection.

Regarding the hyperparameters, the associated priors were chosen to keep the overall between-hosts and between-genotypes variance low. This is indeed a sensitive choice, which may impacts the quantiles of infection duration.

To investigate this point, we performed an additional sensitivity analysis, shown in the new Supplementary Figure S7. We see that for the censored follow-ups, the choice of the standard deviation (sd) of the hypeprior parameter affects the infection duration. For the doubly-censored follow-ups, the impact of the value of sd is particularly pronounced.

Following this sensitivity analysis, we wanted to check that our assumptions for the priors were consistent with data for the literature. For this, we performed a prior predictive check, shown in our new Supplementary Figure S16. This was done by simulating 1,000 datasets with the same structure as the PAPCLEAR data, using parameter

values drawn in our priors. The dynamics of HPV infection clearance we obtain are consistent with the data from Insinga *et al.* (2010) for oncogenic HPV genotypses.
In the revised version, we now further detail the impact of the choice of the priors in general. As expected, some have a strong impact on the model result. This is the case for ξ, the error on the estimate of the time alignment parameter, because the posterior necessarily follows the priors for all the doubly censored follow-ups. Others are less sensitive, as illustrated by Figure S5, which shows the distribution of priors and posteriors of the parameters governing the viral kinetics model.
Overall, in this revised version of the viral kinetics model, the sensitivity of each parameter to the result is clearer thanks to our improved incorporation of the prior information on data censorship. Furthermore, it enables us to use a log-normal distribution for all durations, and we therefore do not have to make arbitrary choice about the maximal infection duration, as was made previously. As expected, there remains an important impact of the priors governing ψ_{t0}, as the model cannot inform this parameter when the follow-up is doubly censored, and a lesser impact of the priors on the parameters governing the infection duration itself, as well as the hyperpriors of the random effects governing the infection duration.

=> We improved our statistical model by improving the incorporation of information about data censorship and by removing the strong hypothesis of maximal infection duration. We also added a new Figure S16 for the prior predictive check, a new Figure S7 for the sensitivity analysis of the choice of hyperpriors, and now further discuss the role of the priors in general in the Discussion.

Q 1.2 *For the viral load kinetic model presented in Figure 4, is there a simpler baseline model (from either the literature or from the authors' own experience) with fewer parameters, over which the proposed model shows improvement? Similarly, is there a quantifiable improvement of the three-step over the two-step model or is this only displayed visually in Figure S6?*

Reply: In general, we are not aware of earlier empirical work following HPV virus load in non-persisting infections. The few existing studies have been conducted in older women, on chronic infections, and usually with a single time point per year. As a consequence, there are also very few mathematical models, besides the one developed by our team and from which our three slopes model is inspired.
As pointed out by the Reviewer, we also analyse a second model with fewer parameters. This simpler model has only an exponential growth phase and an exponential decay phase, as many virus kinetics models. In addition to Figure S7, we also perform a leave-one-out (LOO) cross-validation. The main piece of information comes from the fact that it is difficult to get the model without the plateau to converge, except with exaggeratedly long infections.

=> We clarified the state of the literature and the lack of empirical or mathematical modeling studies on HPV kinetics.

Q 1.3 *In Figure 2B, the authors look for associations between cytokines and cell clusters, though these data appear to combine both infection and uninfected samples. It would be informative to repeat this analysis after splitting the data into focal and non-focal subsets as this may improve resolution of cell-cytokine associations specific to focal infections.*

Reply: We thank the reviewer for this suggestion! In the revised version, we now restrict Figure 2B to HPV focal infections and show in Supplementary Figure S4 a similar correlation matrix between cell populations and cytokines in the case of HPV-negative samples. This allows us to identify a correlation between IFNγ with both monocytes, CD4, and CD8 T cells, that appears to be specific to HPV focal infections.

=> We focused Figure 2B on HPV focal infection samples and added a new Figure S3 for HPV negative samples.

Q 1.4 *In a subset of participants in which sampling prior to, during, and following HPV infection are available, it would be informative to perform a functional flow cytometry assay by stimulating collected cells with HPV peptides or inactivated whole virus then determining cytokine production by ICS - i.e. determine the cellular*

source of the cytokines. However, I appreciate that this may not be possible with the limited cells obtained from cervical sampling. To get around this issue (and if additional funds are available), the authors should consider single-cell RNA sequencing as another option to determine the cellular source of cytokines, which would also detect some of the additional cell types (e.g. ILCs) hypothesized to be present in the Discussion.

Reply: These are all analyses that we would have loved to be able to perform but that were too challenging based on the state-of-the-art at the time the study was conducted (between 2016 and 2020) and because of technical constraints.
As we now further clarify in the manuscript, the flow cytometry analyses were performed on cervical smears, which is very challenging as illustrated by the relative paucity of such studies. Indeed, cervical smears are not ideal tissues. They are fragile and need to be processed fresh (freezing the sample was not an option). Furthermore, most of the cells in the tissue are not leukocytes but keratinocytes, which are large, adhesive, and auto-fluorescent cells — three properties that any flow cytometry platform will dislike. In fact, we only succeeded in identifying the few live leucocytes in this dataset because we automatised the whole pipeline and performed unsupervised clustering.
Even if we do not have samples allowing such analyses, we now reflect in the manuscript on how such an analysis could be done. The literature has (very) few examples of single-cell transcriptomics analyses from cervical cells but they all seem to be based on biopsies. This is an issue for longitudinal follow-ups: biopsies are more invasive than smears and performing them on a bi-monthly basis in healthy volunteers (since an HPV infection is usually benign in the studied population) would raise strong ethical issues. Perhaps this could be achieved with cervical smears but developing an adequate protocol would represent a challenging project in itself. An intermediate step, which we now mention in the manuscript, would be to perform a more thorough analysis of the populations present in the cervical environment. For this, mass cytometry (CyTOF) could represent a particularly interesting option since it would be less sensitive to fluorescence issues. For example, this could generate testable hypotheses for transcriptomics analysis or help with the deconvolution of the transcriptomics data to confirm the presence of particular cell populations. Another option could be to use single-cell multiomics approaches (e.g. BD Rhapsody system) that combine transcriptomic and proteomic analyses. However, setting it up would represent a project in itself in particular to master vaginal smears and their unusual tissue nature (large and adhesive cells).

=> We added a paragraph in the Introduction to rapidly clarify the hurdles raised by the biological system studied. We also now discuss concrete applications to go towards single-cell -omics data in cervical smears.

Minor Comments

Q 1.5 *'Longitudinal cohort' is redundant as 'cohort' implies longitudinal sampling is performed*

Reply: We now use the term longitudinal study to avoid redundancy.

Q 1.6 *Please include a complete flow cytometry gating scheme with representative images of each cell cluster in supplementary materials*

Reply: As we indicate in the manuscript, our identification of the cell populations was done in two ways. First, we identified leucocytes (i.e. CD45+ cells). Second, we identified cell cluster among these leucocytes. In both cases, these analyses were done automatically through unsupervised clustering. Put differently, the algorithm analysed all the information from the cell structure (FSC, SSC) and the 10 fluorescent markers simultaneously. Finally, all the data from all the participants was analysed simultaneously.
Based on this, it would make no sense to perform a manual gating on a handful of carefully selected samples to have beautiful illustrations.
To make the cluster identification from the FlowSOM clustering algorithm more transparent, we added the new Supplementary Figure S2, which shows the fluorescent signal distribution for each individual cluster. Practically, we only used Figures S1 and S2 to merge and annotate the clusters so we think this should allow the reader to gain

more insight into the process. This complements the Supplementary Figure now labelled as S3, which shows the signal intensities on the UMAO clusterings.

=> We now refer to the new Supplementary Figure S2, along with Figures S1, S3, and Table S2 in the main text.

Q 1.7 *Table S1 is missing (labeling begins at Table S2)*

Reply: We apologize for the typo, which is corrected in the revised version.

Q 1.8 *In Fig 1B and C, please align Groups I-XI*

Reply: This has been corrected in the revised version.

Q 1.9 *Data from the multivariate model is not present in Fig. 1C (line 70)*

Reply: We were unsure about how to read this comment so we hope our answer is not too off. Our interpretation is that this is a concern about the lack of details regarding the raw results. Therefore, we now present all the outcomes of the differential analysis with the diffcyt R package in Table S2. As we mention in the manuscript, our decision to use this package rather than perform a classical generalised linear mixed model was guided by the nature of the data, which is compositional and highly dimensional. diffcyt implemented state-of-the-art methods (at least at the time the analysis was performed) for differential abundance analysis in the flow cytometry community.
Furthermore, working on addressing this comment led us to realise that diffcyt differential analysis is not well suited for multivariate analysis. Therefore, we now perform two separate models, one stratifying on HPV infection status and the other stratifying on HPV vaccination status. The two exhibit different patterns in terms of immune cell population shifts. These results are now included in the Supplementary Materials in Table S4.

=> We added a Supplementary Table S3 describing the details of the multivariate model from Figure 1C and performed a separate analysis to detect potential associations between cell populations and vaccination status (shown in Table S4).

Q 1.10 *Please order and align cytokines in Fig 2A and 2B to match*

Reply: This has been corrected in the revised version.

Q 1.11 *Fix typo in line 85 from "0.96" to "0.096"*

Reply: This has been corrected in the revised version.

Q 1.12 *Figure 8 legend references "Fig 7C", which does not exist*

Reply: We corrected the typo in the revised version (it should have read Figure 6A).

Q 1.13 *Was there a blocking step included in the flow cytometry staining methods?*

Reply: There was no blocking step before the flow cytometry staining. This was envisaged in pilot studies but the cell yield after our protocol was sometimes low. Adding a blocking step would have added an additional washing step to the protocol, thereby further reducing the cell yield. Therefore, and also given the stringent cleaning steps involved in the unsupervised pipeline, we chose to bypass this optional step.

=> We now explicitly mention in the Methods of the main text the absence of blocking step.

Q 1.14 *Please define all model terms (e.g. $\psi Pmax$ and ξPj are undefined)*

Reply: In the revised version, we paid particular attention to this issue and made sure that all the parameters were defined in tables S10, S11, S12.

Q 1.15 *Fig S10 color scale difficult to visualize; could include a light grid*

Reply: We added a light grid to improve the visualisation of the figure, now labelled as Fig. S12.

Q 1.16 *Fig S12 appears to have missing values for 'x'*

Reply: We apologise for the mistake, which we corrected in the figure now labelled as Fig. S15.

Q 1.17 *It is unclear if cytokines and antibodies were measure from peripheral blood or from cervical samples. Please indicate*

Reply: Cytokines were measured from cervical secretions but antibodies were measured from peripheral blood. As we now further explain in the revised version, this choice was motivated by the fact that we did not expect HPVs to be immunogenic enough to induce a cytokine response detectable in the bloodstream. Regarding the antibodies, there is a lot of debate in the field about the relevance of local titers whereas systemic HPV antibodies are a well-recognised marker of previous infection with clear threshold values for seropositivity.
=> We made sure to clarify the sampling compartment for the immune-related variables (cytokines, antibodies, and immune cells).

Q 1.18 *After 'samples with visible pellets' are removed, how many remain for analysis?*

Reply: From 632 acquired samples, 161 (25.5%) displayed visible red blood cells pellets. Among the 461 samples remaining, 68 did not pass quality controls using the PeacoQC R package (14.8%). We eventually analysed 393 samples in this study. 202 of these were positive for an HPV focal infection in the clustering analyses (Figure I and II). 233 of the samples analysed were positive for any HPV (focal or non-focal) for the longitudinal analysis (Figures III to VI). Finally, 160 samples were negative for any HPV.

=> We now indicate clearly the number of each flow cytometry samples analysed at each step.

Q 1.19 *Table S5: it would be informative to include the 'Explained Variance' metric from the brms R package*

Reply: We added an estimation of the explained variance (Bayesian R^2) for the brms model in Figure 2A in Table S7.

Q 1.20 *Line 212, there is a space needed after TCR*γδ

Reply: This has been corrected.

Reviewer 2

This is an impressive paper analyzing unique and precious longitudinal data on HPV infectious in a large group of healthy volunteers. The data provides a first look into the typical duration of such infections, the frequency with which they occur and the type of immune responses they invoke. The data is skillfully analyzed with appropriate piecewise models and fitted using a mixed effect population approach using Bayesian approaches in Stan. Because HPV infections occur so frequently, because they have very important health consequences, because they are only cleared after a very long period, and because the analysis is cutting-edge, this manuscript was an exciting read.

Reply: Thank for these very kind words!

That being said my major recommendation would be to improve the readability of the paper. Keep the general audience in mind. The methods are complicated and novel, and the writing style is quite technical, e.g., consistently speaking of clusters rather than cell types, technical abbreviations like "clr", and sometimes complicated wording like "which is consistent with the hypothesis of a biological mechanism" (line 281). I admire the non-supervised definition of cellular phenotypes, but the reader would be helped when the biological interpretations of the 11 clusters are repeated more frequently.

Reply: We agree with the reviewer on this point, especially since, as we explained in detail in the cover letter, it may have led to some misunderstandings about what is doable in such a biological system.

=> We took particular care to make the first three sections (Introduction, Results, and Discussion) accessible to an audience as wide as possible. This was done by clarifying the general setting (e.g. why studying HPV kinetics is so challenging), trying to avoid technical terms as much as possible, and explaining more intuitively why the different analyses were done.

It would also be good to expand the biological discussion:

Q 2.1 *What is the effect on vaccination on infections with other HPV genotypes?*

Reply: We did not detect any 'breakthrough' infection (i.e. a vaccinated host infected by a vaccine type) but vaccination against HPV is one of the factors we looked at in the first place in the hope of finding signs of cross-immunity.
Part of the answer is provided in the inclusion data, which was reported in a previous study (Murall *et al.* 2020 *Vaccine*). There, we find that HPV51, which is not included in any vaccine, is less prevalent in vaccinated women, a finding already reported in at least one cohort.
Here, we could go further thanks to the longitudinal analyses and the immunological data. Overall, the effects are a bit disappointing as vaccination does not come out of any of our models. As shown in Fig. 8B, there is a trend for vaccinated participants to have milder infections with smaller viral load AUC, but this effect was not significant.

=> We now added subsections to the Discussion and focused one of these on vaccination to better discuss this issue.

Q 2.2 *Why do you think it takes so long to clear an infection while the innate responses start quite early?*

Reply: As noticed by Reviewer #1, the level of accuracy we can reach with our immunological data is limited. In our data, the innate immune response seems to be stronger in infections that clear rapidly, whereas the adaptive immune response is higher in infections that last longer. Our current interpretation of this observation is that the innate immune response is activated in all women upon infections but it does not always succeed in clearing the infection, in which case the adaptive immune response kicks in. Therefore, the presence of a stronger adaptive immune response could be an indication of the inability of the immune system to clear the infection rapidly. This is also consistent with the known fact in the HPV literature that only half of the infections lead to seroconversion.

=> We now highlight our general interpretation of the immune dynamics (an issue also raised by Reviewer #3) in the Discussion.

Minor recommendations

Q 2.3 *I am confused that the initial viral load, v_0, is identifiable parameter because the time of infection is not known. Wouldn't the Viral load growth duration define both the time of infection and the initial viral load?*

Reply: Thank you for pointing this out. In the new version of our model, this parameter v_0 is now fixed at the lowest possible limit of detection of our qPCR. This limit depends on the quality of the sample, measured by the number of albumin copies, and has a minimum of 10^{-4} copies/cell following the literature.

Q 2.4 *I would provide a reference to FlowSOM and provide a short sentence explaining its algorithm.*

Reply: This has now been corrected.

Q 2.5 *line 50 "We could also notably identify" => We identified*

Reply: This has been corrected in the revised version.

Q 2.6 *line 145 Why would baseline values of cytokines be correlated?*

Reply: We assumed that the baseline variation between women could be driven by physiological differences such as BMI, stress, or nutrition. By all affecting the local level of inflammation, they could be jointly affecting the baseline level of the local immune markers that we measured.
=> We added these details in the methods section.

Q 2.7 *In Fig 7 The shading of the bullets indicates total protein or total cell numbers. Although it is excellent that this information is provided, it quite difficult to see*

Reply: In the revised version of the figure, we added two lines, one indicating the total protein concentration, and the other the total number of leukocytes in each sample. We further stress that these are indicators of the quality of the cytokines and flow cytometry measurements respectively.

Q 2.8 *line 543 "Still notation wise, in equation system" => In equation system*

Reply: We changed the formulation accordingly.

Reviewer 3

This is a very interesting article on the natural history and immune dynamics of HPV infections, based on a unique cohort of healthy women sampled every two months over a relatively long time period. The paper is well written (perhaps at times a bit difficult to read; but this is to some extent inevitable given the complexity of the topic) and the analysis seems sound. I particularly liked the idea of exploring "the frontier between acute and chronic infections " which I expect to be of broader interest.

Reply: Thank you for the positive appreciation of our work!

I have the following suggestions:

Q 3.1 *It would be interesting if the authors could discuss the generalizability of their findings beyond the specific population included.*

Reply: The reviewer is correct in that the population we follow is very specific, most of the participants being students in France, a country with good universal health coverage.
Regarding the kinetics themselves, since this is the first study of its kind, we cannot use any prior knowledge. However, as we mention in the article, it is interesting that the observed pattern matches the outcome of a conceptual model using parameter values inspired from the literature.
A more concrete way to position our results in a broader context is to note that the type of cell populations we identify by flow cytometry are consistent with other studies performed on similar tissues (although on different populations). This is in particular the case for the dominance of granulocytes among leucocytes (Mohd Zaki *et al*, *Front Immunol*, 2022). We also observe a consistent order of accumulation of leukocyte cells throughout the

infection, starting with neutrophils, and ending with cells classically identified as involved in the adaptive immune response (i.e. CD4 and CD8 T cells). Furthermore, the prevalence of the HPV types in our study is consistent with what can be expected for Europe.

=> In the Discussion, we added a section to recast our findings in a broader context and elaborate on the specificity of the population we analyse.

***Q* 3.2** *The authors fit the virus load dynamics with a phenomenological/statistical model consisting of an increasing slope, a plateau, followed by a decreasing slope. It would be good if they could provide some mechanistic justification for this assumption (as it is interesting but rather unusual). In the discussion they refer to a previous paper ("Building on an existing mathematical model [32] and prior knowledge on infection duration [7], we show that HPV infections can be summarised in three phases... ") as a potential explanation but it would be good to have this reasoning earlier and more explicitly in the manuscript. Similarly, it was not fully clear to me how "This assumption regarding the shape was motivated by the high frequency of left- and right-censored follow-ups ... ". Please clarify.*

Reply: As already pointed out by the reviewers, this is one of the instances where our formulations were not detailed enough in the initial version and we expanded on this.
As summarised by the reviewer, there were two main motivations to try the three slopes model.
The first one is our previous mathematical model. Although we should have come up with this ourselves, to be honest, this suggestion was made by someone from the audience when one of us presented this model and then the clinical data (which we were analysing with 2 slopes at the time). We followed the suggestion and it turns out the fit was much better. In the revised version, we now explain why such a trend emerges from the mechanistic model. In short, the driver is the stratified nature of the epithelium infected by HPVs, which resembles an age structure in ecology. For viruses causing more systemic infections, such as hepatitis C virus or HIV, the target cell population is much more homogeneous, which leads to classical acute infection patterns (an exponential increase and an exponential decrease). To make a parallel with ecology, in the latter case, the resource is used all at once. Therefore, the three slopes seem to originate from the fact that HPVs follow the life cycle of the epithelial cells and from the low immunogenicity of the infection. Importantly, these insights were generated by a conceptual model. Although we took particular care in setting up the general structure and choosing relevant parameter values, there was no data to compare it to.
The second motivation for introducing a plateau is less theoretical and more empirical: the raw data of the individual follow-ups do not seem consistent with a two-slope (acute) infection model. From a statistical standpoint, this is particularly penalising when attempting to fit truncated follow-ups, in which the growth phase and/or clearance phase are not observed.

=> We now introduce the idea of the three slopes from the Introduction, by discussing our earlier mathematical model. Then, in the Results section, we clearly state why we opted for this model.

***Q* 3.3** *The same applies to some extent also to the two-slope model for the immune dynamics. Here, it would be additionally good if the authors could argue to what extent this qualitatively matches the observed dynamics.*

Reply: One important difference between the immune dynamics and the viral dynamics is that for each analyte there is only a single time series per participant, whereas several participants have more than one virus load time series. Therefore, the immune kinetics we describe are a global response to all HPV infections occurring in the host.
Also contrarily to the virus kinetics, there was no a priori reason to expect a plateau for immune cell population densities or cytokine concentrations. Furthermore, the raw data data did not support a three slopes model. Finally, the fact that many participants are co-infected already yields non-trivial patterns of the immune cells populations due to our additive assumption.
Overall, we opted for the most parsimonious and biologically-relevant approach for the immune response with only an activation (growth) phase and a deactivation (decrease) phase.

=> We now clearly justify the choice of a two-slope model for immune dynamics.

Q 3.4 *It would be good if the authors could provide a bit more interpretations/speculations/context for the different immune markers they find to be associated with HPV infection (or infection characteristics); e.g. what does the association of TCR-gamma-delta cells and CXCL10 concentration with HPV infection imply for our understanding of HPV immune dynamics.*

Reply: We tried to give more details but these tend to be more hypotheses than interpretations. Part of the limit comes from the fact that the quality of our longitudinal follow-up had to be traded off against the accuracy of our description of the immune population.
One of the illustrations of these limitations is that we are able to identify a cellular population (cluster VII) with a potentially important role through unsupervised clustering. However, due to the lack of markers, we are constrained to reason by elimination and are eventually unable to provide their formal identification. In the end, we cannot rule out two populations, B cells from ILCs or immune progenitors.
In general, we think our results should be taken as maps for future research in that they indicate in which direction to look. CXCL10 is a cytokine involved in the inflammation process which has been associated with several infections, such as HCV and HIV, but also more recently HPV (Usyk *et al.* 2020). As the product of an interferon-stimulated gene, it is a marker of ongoing anti-viral responses, which promotes effector T cell recruitment through CXCR3. This makes our results consistent with previous findings, reinforcing the robustness of our analyses.
The identification of TCR-gamma-delta T cell subsets as immune correlates of HPV infection is also interesting because these cells have characteristics from both the innate and the adaptive immune response, and play a crucial role in epithelial barrier defences. Our results, in general, suggest an involvement of both these responses — our general interpretation being that in some infections the local response is sufficient to achieve rapid clearance and that in others the adaptive immune response is triggered with the recruitment of additional cell subsets upon CXCL10 production. A better understanding of which TCR-gamma-delta populations are involved could help understand this dual pattern. Furthermore, that some of these cells are known to play a particularly important against cancers echoes the carcinogenic properties of HPVs. In fact, one of our consortium teams identified a role for TCR-gamma-delta cells in HPV-induced cancers. Overall, TCR-gamma-delta cells could be the key to determining whether HPV infections are cleared (i.e. 'acute') or become persistent, the latter being at risk of evolving into HPV-induced cancers.

=> We now extrapolate on the immunological implications of our results. In particular, we highlight the ability of unsupervised clustering to identify key populations even if they cannot be clearly labelled (e.g. cluster VII). Furthermore, as indicated in our response to Reviewer #2, we provide a more global interpretation of our results in terms of HPV rapid or slow clearance by the immune response.

Q 3.5 *Could these immune markers (TCR-gamma-delta cells and CXCL10) be corroborated in women with incident infections or in women clearing HPV infections (i.e. do they increase/decrease upon infection or clearance in the way predicted by their overall associations with HPV status across individuals).*

Reply: Such an analysis cannot be robustly performed with our current data, but this is indeed the trend we detect in our cohort so corroborating it with another dataset would undoubtedly be of interest.
A cohort similar to ours could be set up again (since we succeeded in doing it) but we are not aware of existing datasets that could allow for a rapid validation. The difficulty, which explains why we know so little about the immune response to non-persisting HPV infections, is that few studies have frequent enough follow-ups to ensure that an absence of detection is indeed a clearance or that a re-detection is not a re-infection by the same genotype. Furthermore, many of the HPV cohorts only perform genotyping and do not collect immunological data (especially flow cytometry data).
We now mention the difficulty of validating these findings with existing data. We also further describe two studies that found consistent results. Shannon *et al.* (2017) found higher CXCL10 titers in persistent HPV infection, but no association with 'clearance', which they define as the absence of detection of any HPV 6 months later.

Second, Usyk *et al.* (2022) found that higher titers of CXCL10 are associated with reduced 'clearance'. As in the previous study, they defined clearance as the absence of *any* HR-HPV (whereas we work at a genotype-specific level). Furthermore, the average time between the collection of two samples was 1.28 years.
Overall, we are not aware of existing data involving TCR-gamma-delta cells but other studies suggest associations with CXCL10 and HPV infections. It is difficult to go further because different studies do not define clearance (or even infections) in the same way.

=> We better underline the challenging of comparing our results to existing ones given the limitation of existing cohorts in terms of follow-up density and/or availability of flow cytometry data.

Q 3.6 *Also it was not fully clear to what extent the different immune markers can explain the clearance after the end of the plateau.*

Reply: We voluntarily did not emphasize the clearance as such in terms of process because many of our follow-ups are truncated. Therefore, we are confident that we have the statistical power to comment on the duration of the infection (or the total amount of viruses produced, which is related to it) but less to provide mechanistic insights about the clearance itself.
=> We now mention our lack of statistical power to make mechanistic inferences about HPV clearance.

Q 3.7 *I would rather omit the sentence "Our linear models identified positive correlations between the 83 concentration of IFNγ and the frequency of CD8 T cells (cluster XI, with a regression coefficient, see the Methods, β = 0.11), although with a p-value of 0.96." given the p-value.*

Reply: We completely agree and would never have written this ourselves voluntarily. It turns out there was a typo and the p-value is 0.096, which is above the 5% threshold but is nevertheless quite low for such an observational clinical study.

=> This was corrected and the new Figure 2B is also now restricted to HPV focal infections.

Q 3.8 *Given the large number of different often non-standard analyses performed, it would be good if the code could be provided in a repository (e.g. githbub or similar).*

Reply: Yes, the code along with the raw data will be shared upon publication (hopefully sooner). We are polishing the scripts to make sure they are understandable. We are happy to share it at this point if it can help the reviewing process.

Q 3.9 *The sentence "Our virus kinetics model assumes that the infections are independent. Therefore, we aligned each time series to the time to the observed midpoint of the corresponding infection " was not fully clear to me. Please clarify.*

Reply: This comment got us thinking and we realised there was a way to improve the statistical model using some information we were neglecting in the original version.
First, instead of using a random effect to compute ψ_{t0}, we use a fixed effect for each infection, which implies independence between the date of each infection. In particular, this implies assuming no particular risk of co-acquisition of several genotypes at the same time by the same individual.
Second, as explained previously, we now treat differently the computation of ψ_{t0} depending on the follow-up censoring status, in order to better incorporate the uncertainty in the estimation of this parameter into the prior distribution. Indeed, we are much more uncertain of the date of infection when the follow-up is doubly censored than when we observe at least the date of infection or clearance.
