# Supplementary material for: Viral and immune dynamics of genital human papillomavirus infections in young women with high temporal resolution
Source: PLoS Biol. 2025 Jan 21;23(1):e3002949. doi: 10.1371/journal.pbio.3002949 (PMC11750104; doi:10.1371/journal.pbio.3002949)
Supplement: Attachment — Submitted filename: response_rev_plosbio_R2.pdf [file pbio.3002949.s003.pdf]

Replies to the reviewers

Reviewer 1

In this revised version of the article "Viral and immune dynamics of human papillomavirus genital infections in young women", Tessandier and Elie et al. make appreciable updates to the study in response to the reviewers. Notably, the authors improved the statistical model by incorporating information about data censorship, removing the hypothesis of maximal effect duration, and including a sensitivity analysis related to the choice of priors. The authors also included an additional analysis of cell and cytokine associations broken down by infection status, identifying a new association with interferon gamma. Lastly, the authors include updates to the text regarding specific technical limitations and future directions such as CyTOF. That being said, the text is quite long and detailed. Some of the explanations of the model (or the validation of various aspects) could be moved into the methods text constrains become an issue. Overall, this exciting study has been improved by the authors' responses to the reviewers and this important work warrants publication.

Reply: Thank you again for your careful reading and the appreciation of our manuscript!

Reviewer 2

NA

Reviewer 3

The authors have addressed my comments very well.

Reply: Thanks!
